# *catena*-Poly[[dichloridozinc(II)]-μ-1,1′-(butane-1,4-di­yl)diimidazole-κ^2^ *N* ^3^:*N* ^3′^]

**DOI:** 10.1107/S1600536810018246

**Published:** 2010-06-05

**Authors:** Ren-Ling He, Fan-Jin Meng, Guan-Hua Wang, Wei Yang, Jing-Wei Xu

**Affiliations:** a The State Key Laboratory of Electroanalytical Chemistry, Changchun Institute of Applied Chemistry, Chinese Academy of Sciences, Changchun 130022, People’s Republic of China

## Abstract

The title compound, [ZnCl_2_(C_10_H_14_N_4_)]_*n*_, is a coordination polymer consisting of zigzag chains propagating in [001], in which the metal cation exhibits a distorted tetrahedral ZnCl_2_N_2_ coordination. Adjacent chains are linked by inter­molecular C—H⋯Cl hydrogen bonds, forming a three-dimensional supra­molecular network.

## Related literature

For general background to metal complexes of *N*-heterocyclic compounds, see: Hu *et al.* (2003 ▶); Ohmori *et al.* (2005 ▶); Chen *et al.* (2004 ▶); Hu *et al.* (2005 ▶). For related structures, see: Li *et al.* (2006 ▶); Liu *et al.* (2007 ▶); Jin *et al.* (2007 ▶); Yang *et al.* (2009 ▶); Qi *et al.* (2008 ▶).
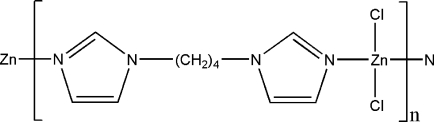


## Experimental

### 

#### Crystal data

[ZnCl_2_(C_10_H_14_N_4_)]
*M* *_r_* = 326.52
Monoclinic, 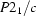

*a* = 7.8090 (9) Å
*b* = 11.6001 (13) Å
*c* = 15.8047 (18) Å
β = 92.908 (2)°
*V* = 1429.8 (3) Å^3^
*Z* = 4
Mo *K*α radiation
μ = 2.08 mm^−1^
*T* = 185 K
0.29 × 0.22 × 0.15 mm

#### Data collection

Bruker SMART APEX CCD diffractometer
Absorption correction: multi-scan (*SADABS*; Sheldrick, 1996 ▶) *T*_min_ = 0.585, *T*_max_ = 0.742
7827 measured reflections
2820 independent reflections
2174 reflections with *I* > 2σ(*I*)
*R*_int_ = 0.054

#### Refinement

*R*[*F* ^2^ > 2σ(*F* ^2^)] = 0.051
*wR*(*F* ^2^) = 0.107
*S* = 1.06
2820 reflections
154 parameters
H-atom parameters constrained
Δρ_max_ = 0.68 e Å^−3^
Δρ_min_ = −0.36 e Å^−3^

### 

Data collection: *SMART* (Bruker, 2007 ▶); cell refinement: *SAINT* (Bruker, 2007 ▶); data reduction: *SAINT*; program(s) used to solve structure: *SHELXS97* (Sheldrick, 2008 ▶); program(s) used to refine structure: *SHELXL97* (Sheldrick, 2008 ▶); molecular graphics: *SHELXTL* (Sheldrick, 2008 ▶) and *Mercury* (Macrae *et al.*, 2006 ▶); software used to prepare material for publication: *SHELXTL*.

## Supplementary Material

Crystal structure: contains datablocks global, I. DOI: 10.1107/S1600536810018246/ez2200sup1.cif

Structure factors: contains datablocks I. DOI: 10.1107/S1600536810018246/ez2200Isup2.hkl

Additional supplementary materials:  crystallographic information; 3D view; checkCIF report

## Figures and Tables

**Table 1:** Hydrogen-bond geometry (Å, °)

*D*—H⋯*A*	*D*—H	H⋯*A*	*D*⋯*A*	*D*—H⋯*A*
C3—H3⋯Cl1^i^	0.93	2.63	3.538 (2)	166
C5—H5⋯Cl2^ii^	0.93	2.78	3.599 (5)	147

Symmetry codes: (i) 
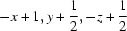
; (ii) 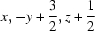.

**Fig. 1.**
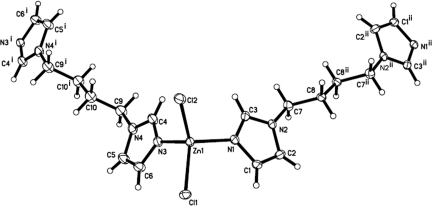
Partial molecular structure of the title compound showing the atom numbering scheme. Displacement ellipsoids are drawn at the 30% probability level. Atoms marked with i or ii are at the symmetry positions (x+2, y+5/2, 3/2-z) and (x, y+5/2, 1/2-z) respectively.

**Fig. 2.**
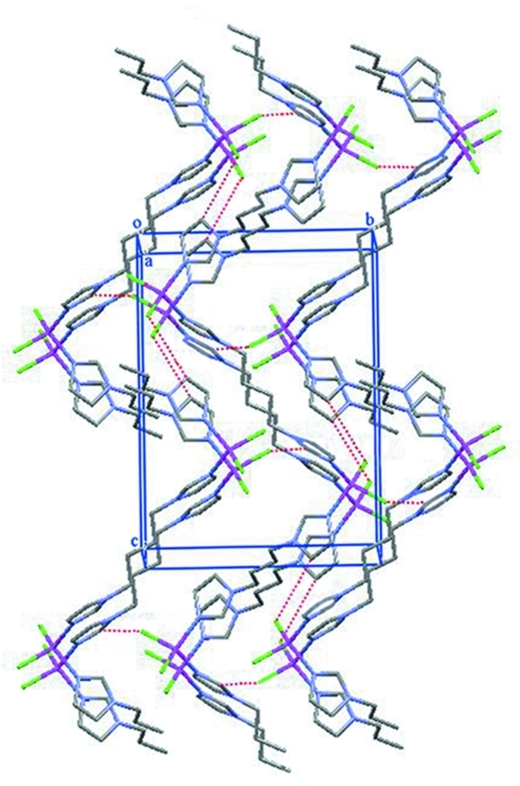
The zigzag polymeric chain structure of the title compound. Dashed lines denote hydrogen bonds.

## supplementary crystallographic information

### Comment

N-heterocyclic compounds have been extensively studied in coordination chemistry research for their excellent bridging ability (Hu *et al.*, 2003; Ohmori *et al.*, 2005; Chen *et al.*, 2004; Hu *et al.*, 2005). The compound 1,1'-(1,4-butanediyl)bis(imidazole) (bbi), as a flexible nitrogenous ligand with a long -CH_2_CH_2_CH_2_CH_2_- spacer, can link discrete clusters into an extended network and is a good candidate to form highly connected 3D frameworks. A number of metal-bbi coordination polymers have been reported (Li *et al.*, 2006; Liu *et al.*, 2007; Jin *et al.*, 2007; Yang *et al.*, 2009; Qi *et al.*, 2008). Here we present a new polymeric compound, [ZnCl_2_(bbi)]_n_, (I), with a zigzag chain structure, synthesized under solvothermal conditions.

In the title compound, (I), the Zn centers are four-coordinated by two N atoms from two bbi ligands [Zn(1)—N(1) = 2.005 (3) Å and Zn(1)—N(3) = 2.013 (3) Å] and two Cl atoms [Zn(1)—Cl(1) = 2.2557 (11) Å and Zn(1)—Cl(2) =2.2321 (12) Å], resulting in a distorted tetrahedral geometry (Fig. 1). Each bbi coordinates to two Zn atoms through its two aromatic N atoms and acts as a bridging bidentate ligand to form a one-dimensional zigzag chain (Fig. 2). The adjacent Zn···Zn distance is 14.290 Å, which is similar to that observed in [Cu_2_(bbi)_2_Cl_2_] (Qi *et al.*, 2008). In addition, these one-dimensional chains are further connected by weak intermolecular C—H···Cl hydrogen bonds to construct a three-dimensional supramolecular network (Fig. 2).

### Experimental

The title compound was solvothermally prepared from a reaction mixture of ZnCl_2_ (0.3 mmol), bbi (0.1 mmol), ethanol (3 ml) and distilled water (7 ml); the pH value was adjusted to 4.5 with triethylamine and acetic acid. The mixture was stirred for 20 min at room temperature, then sealed in a 20 ml teflon-lined stainless steel autoclave and heated at 433 K for 72 h under autogenous pressure. After cooling to room temperature, colorless block crystals were obtained (yield 83% based on Zn).

### Refinement

H atoms were positioned geometrically and refined with fixed individual displacement parameters [U_iso_(H) = 1.2U_eq_(C)], using a riding model, with C—H distances of 0.93 Å for C*sp*^2^ and 0.97 Å for CH_2_.

### Crystal data

**Table:**

[ZnCl_2_(C_10_H_14_N_4_)]	*F*(000) = 664
*M**_r_* = 326.52	*D*_x_ = 1.517 Mg m^−^^3^
Monoclinic, *P*2_1_/*c*	Mo *K*α radiation, λ = 0.71073 Å
Hall symbol: -P 2ybc	Cell parameters from 2763 reflections
*a* = 7.8090 (9) Å	θ = 2.2–26.1°
*b* = 11.6001 (13) Å	µ = 2.08 mm^−^^1^
*c* = 15.8047 (18) Å	*T* = 185 K
β = 92.908 (2)°	Block, colorless
*V* = 1429.8 (3) Å^3^	0.29 × 0.22 × 0.15 mm
*Z* = 4	

### Data collection

**Table:**

Bruker SMART APEX CCD diffractometer	2820 independent reflections
Radiation source: fine-focus sealed tube	2174 reflections with *I* > 2σ(*I*)
graphite	*R*_int_ = 0.054
φ and ω scans	θ_max_ = 26.1°, θ_min_ = 2.2°
Absorption correction: multi-scan (*SADABS*; Sheldrick, 1996)	*h* = −9→9
*T*_min_ = 0.585, *T*_max_ = 0.742	*k* = −11→14
7827 measured reflections	*l* = −19→19

### Refinement

**Table:**

Refinement on *F*^2^	Primary atom site location: structure-invariant direct methods
Least-squares matrix: full	Secondary atom site location: difference Fourier map
*R*[*F*^2^ > 2σ(*F*^2^)] = 0.051	Hydrogen site location: inferred from neighbouring sites
*wR*(*F*^2^) = 0.107	H-atom parameters constrained
*S* = 1.06	*w* = 1/[σ^2^(*F*_o_^2^) + (0.0437*P*)^2^ + 0.2952*P*] where *P* = (*F*_o_^2^ + 2*F*_c_^2^)/3
2820 reflections	(Δ/σ)_max_ < 0.001
154 parameters	Δρ_max_ = 0.68 e Å^−^^3^
0 restraints	Δρ_min_ = −0.36 e Å^−^^3^

### Special details

**Table:**

Geometry. All esds (except the esd in the dihedral angle between two l.s. planes) are estimated using the full covariance matrix. The cell esds are taken into account individually in the estimation of esds in distances, angles and torsion angles; correlations between esds in cell parameters are only used when they are defined by crystal symmetry. An approximate (isotropic) treatment of cell esds is used for estimating esds involving l.s. planes.
Refinement. Refinement of *F*^2^ against ALL reflections. The weighted *R*-factor wR and goodness of fit *S* are based on *F*^2^, conventional *R*-factors *R* are based on *F*, with *F* set to zero for negative *F*^2^. The threshold expression of *F*^2^ > σ(*F*^2^) is used only for calculating *R*-factors(gt) etc. and is not relevant to the choice of reflections for refinement. *R*-factors based on *F*^2^ are statistically about twice as large as those based on *F*, and *R*- factors based on ALL data will be even larger.

### Fractional atomic coordinates and isotropic or equivalent isotropic displacement parameters (Å^2^)

**Table:**

	*x*	*y*	*z*	*U*_iso_*/*U*_eq_	
Zn1	0.46384 (6)	0.61215 (4)	0.27801 (3)	0.02355 (16)	
Cl1	0.33311 (13)	0.46729 (9)	0.34476 (7)	0.0299 (3)	
Cl2	0.67102 (14)	0.55460 (11)	0.19540 (7)	0.0370 (3)	
N2	0.1432 (4)	0.8426 (3)	0.1480 (2)	0.0244 (8)	
N1	0.2770 (4)	0.6993 (3)	0.2141 (2)	0.0249 (8)	
N3	0.5563 (4)	0.7158 (3)	0.3717 (2)	0.0289 (9)	
N4	0.6711 (5)	0.8617 (3)	0.4421 (2)	0.0341 (10)	
C1	0.1030 (5)	0.6818 (4)	0.2160 (3)	0.0319 (11)	
H1	0.0509	0.6195	0.2413	0.038*	
C2	0.0202 (6)	0.7689 (4)	0.1755 (3)	0.0342 (11)	
H2	−0.0980	0.7776	0.1676	0.041*	
C3	0.2941 (5)	0.7974 (4)	0.1726 (3)	0.0283 (10)	
H3	0.3992	0.8307	0.1620	0.034*	
C6	0.5363 (6)	0.6995 (4)	0.4562 (3)	0.0384 (12)	
H6	0.4815	0.6368	0.4796	0.046*	
C5	0.6077 (6)	0.7875 (4)	0.5003 (3)	0.0423 (13)	
H5	0.6131	0.7965	0.5589	0.051*	
C4	0.6379 (6)	0.8141 (4)	0.3661 (3)	0.0359 (11)	
H4	0.6688	0.8468	0.3153	0.043*	
C7	0.1145 (6)	0.9520 (4)	0.1043 (3)	0.0302 (11)	
H7A	0.2243	0.9890	0.0974	0.036*	
H7B	0.0478	1.0018	0.1393	0.036*	
C8	0.0227 (5)	0.9402 (4)	0.0186 (3)	0.0261 (10)	
H8A	−0.0814	0.8957	0.0238	0.031*	
H8B	0.0952	0.8993	−0.0194	0.031*	
C9	0.7655 (6)	0.9682 (4)	0.4623 (3)	0.0438 (13)	
H9A	0.7132	1.0070	0.5089	0.053*	
H9B	0.7574	1.0192	0.4136	0.053*	
C10	0.9514 (6)	0.9456 (4)	0.4862 (3)	0.0423 (13)	
H10A	1.0055	0.9121	0.4380	0.051*	
H10B	0.9591	0.8899	0.5320	0.051*	

### Atomic displacement parameters (Å^2^)

**Table:**

	*U*^11^	*U*^22^	*U*^33^	*U*^12^	*U*^13^	*U*^23^
Zn1	0.0249 (3)	0.0224 (3)	0.0230 (3)	−0.0008 (2)	−0.00252 (19)	0.0021 (2)
Cl1	0.0288 (6)	0.0250 (6)	0.0366 (6)	−0.0026 (5)	0.0069 (5)	0.0052 (5)
Cl2	0.0349 (6)	0.0457 (8)	0.0311 (6)	0.0052 (6)	0.0072 (5)	0.0077 (5)
N2	0.0253 (19)	0.024 (2)	0.0237 (18)	0.0029 (16)	−0.0019 (15)	0.0046 (15)
N1	0.0265 (19)	0.023 (2)	0.0247 (19)	0.0014 (16)	−0.0006 (15)	0.0043 (16)
N3	0.031 (2)	0.028 (2)	0.027 (2)	−0.0034 (17)	−0.0035 (16)	0.0027 (16)
N4	0.039 (2)	0.031 (2)	0.031 (2)	−0.0085 (18)	−0.0086 (18)	−0.0007 (17)
C1	0.028 (2)	0.034 (3)	0.034 (3)	−0.001 (2)	0.002 (2)	0.015 (2)
C2	0.022 (2)	0.038 (3)	0.043 (3)	−0.002 (2)	0.003 (2)	0.012 (2)
C3	0.026 (2)	0.031 (3)	0.028 (2)	−0.003 (2)	−0.0038 (18)	0.004 (2)
C6	0.048 (3)	0.040 (3)	0.028 (3)	−0.018 (3)	0.000 (2)	0.001 (2)
C5	0.054 (3)	0.048 (3)	0.024 (2)	−0.011 (3)	−0.001 (2)	0.001 (2)
C4	0.045 (3)	0.035 (3)	0.027 (2)	−0.007 (2)	−0.006 (2)	0.005 (2)
C7	0.036 (3)	0.022 (3)	0.032 (3)	−0.001 (2)	−0.002 (2)	0.005 (2)
C8	0.024 (2)	0.030 (3)	0.025 (2)	0.005 (2)	0.0043 (18)	0.0050 (19)
C9	0.057 (3)	0.037 (3)	0.037 (3)	−0.018 (3)	−0.007 (2)	0.003 (2)
C10	0.052 (3)	0.034 (3)	0.039 (3)	−0.017 (3)	−0.008 (2)	0.002 (2)

### Geometric parameters (Å, °)

**Table:**

Zn1—N1	2.005 (3)	C3—H3	0.9300
Zn1—N3	2.013 (3)	C6—C5	1.342 (7)
Zn1—Cl2	2.2321 (12)	C6—H6	0.9300
Zn1—Cl1	2.2557 (11)	C5—H5	0.9300
N2—C3	1.330 (5)	C4—H4	0.9300
N2—C2	1.373 (5)	C7—C8	1.506 (6)
N2—C7	1.457 (5)	C7—H7A	0.9700
N1—C3	1.323 (5)	C7—H7B	0.9700
N1—C1	1.375 (5)	C8—C8^i^	1.540 (8)
N3—C4	1.312 (6)	C8—H8A	0.9700
N3—C6	1.364 (5)	C8—H8B	0.9700
N4—C4	1.336 (5)	C9—C10	1.504 (6)
N4—C5	1.369 (6)	C9—H9A	0.9700
N4—C9	1.466 (6)	C9—H9B	0.9700
C1—C2	1.344 (6)	C10—C10^ii^	1.524 (9)
C1—H1	0.9300	C10—H10A	0.9700
C2—H2	0.9300	C10—H10B	0.9700
			
N1—Zn1—N3	107.13 (14)	C6—C5—N4	106.5 (4)
N1—Zn1—Cl2	112.74 (10)	C6—C5—H5	126.8
N3—Zn1—Cl2	111.43 (11)	N4—C5—H5	126.8
N1—Zn1—Cl1	106.02 (10)	N3—C4—N4	111.7 (4)
N3—Zn1—Cl1	104.75 (11)	N3—C4—H4	124.1
Cl2—Zn1—Cl1	114.17 (5)	N4—C4—H4	124.1
C3—N2—C2	106.6 (4)	N2—C7—C8	113.7 (4)
C3—N2—C7	126.6 (4)	N2—C7—H7A	108.8
C2—N2—C7	126.8 (4)	C8—C7—H7A	108.8
C3—N1—C1	105.2 (4)	N2—C7—H7B	108.8
C3—N1—Zn1	126.4 (3)	C8—C7—H7B	108.8
C1—N1—Zn1	127.5 (3)	H7A—C7—H7B	107.7
C4—N3—C6	105.5 (4)	C7—C8—C8^i^	110.6 (5)
C4—N3—Zn1	128.8 (3)	C7—C8—H8A	109.5
C6—N3—Zn1	125.6 (3)	C8^i^—C8—H8A	109.5
C4—N4—C5	106.5 (4)	C7—C8—H8B	109.5
C4—N4—C9	128.0 (4)	C8^i^—C8—H8B	109.5
C5—N4—C9	125.3 (4)	H8A—C8—H8B	108.1
C2—C1—N1	109.3 (4)	N4—C9—C10	112.1 (4)
C2—C1—H1	125.3	N4—C9—H9A	109.2
N1—C1—H1	125.3	C10—C9—H9A	109.2
C1—C2—N2	106.9 (4)	N4—C9—H9B	109.2
C1—C2—H2	126.5	C10—C9—H9B	109.2
N2—C2—H2	126.5	H9A—C9—H9B	107.9
N1—C3—N2	112.0 (4)	C9—C10—C10^ii^	112.8 (5)
N1—C3—H3	124.0	C9—C10—H10A	109.0
N2—C3—H3	124.0	C10^ii^—C10—H10A	109.0
C5—C6—N3	109.7 (4)	C9—C10—H10B	109.0
C5—C6—H6	125.1	C10^ii^—C10—H10B	109.0
N3—C6—H6	125.1	H10A—C10—H10B	107.8

Symmetry codes: (i) −*x*, −*y*+2, −*z*; (ii) −*x*+2, −*y*+2, −*z*+1.

### Hydrogen-bond geometry (Å, °)

**Table:**

*D*—H···*A*	*D*—H	H···*A*	*D*···*A*	*D*—H···*A*
C3—H3···Cl1^iii^	0.93	2.63	3.538 (2)	166
C5—H5···Cl2^iv^	0.93	2.78	3.599 (5)	147

Symmetry codes: (iii) −*x*+1, *y*+1/2, −*z*+1/2; (iv) *x*, −*y*+3/2, *z*+1/2.
